# Gold@silver bimetal nanoparticles/pyramidal silicon 3D substrate with high reproducibility for high-performance SERS

**DOI:** 10.1038/srep25243

**Published:** 2016-05-04

**Authors:** Chao Zhang, Shou Zhen Jiang, Cheng Yang, Chong Hui Li, Yan Yan Huo, Xiao Yun Liu, Ai Hua Liu, Qin Wei, Sai Sai Gao, Xing Guo Gao, Bao Yuan Man

**Affiliations:** 1 School of Physics and Electronics, Shandong Normal University, Jinan 250014, China; 2 State Key Lab of Crystal Materials Shandong University, Jinan 250100, China; 3 School of Science, Qilu University of Technology, Jinan 250353, China

## Abstract

A novel and efficient surface enhanced Raman scattering (SERS) substrate has been presented based on Gold@silver/pyramidal silicon 3D substrate (Au@Ag/3D-Si). By combining the SERS activity of Ag, the chemical stability of Au and the large field enhancement of 3D-Si, the Au@Ag/3D-Si substrate possesses perfect sensitivity, homogeneity, reproducibility and chemical stability. Using R6G as probe molecule, the SERS results imply that the Au@Ag/3D-Si substrate is superior to the 3D-Si, Ag/3D-Si and Au/3D-Si substrate. We also confirmed these excellent behaviors in theory via a commercial COMSOL software. The corresponding experimental and theoretical results indicate that our proposed Au@Ag/3D-Si substrate is expected to develop new opportunities for label-free SERS detections in biological sensors, biomedical diagnostics and food safety.

Surface enhanced Raman scattering (SERS) is a label-free and highly sensitive analytical tool to fingerprint molecular based on an excellent SERS substrate^1^,^2^,^3^,^4^,^5^. With the assist of electromagnetic mechanism (EM) and chemical mechanism (CM), we can achieve SERS enhancement respectively up to 10^14^ and 10–100^6^. The EM enhancement can be realized by the surface plasmons excited between noble metal particles (such as Ag, Cu and Au) by visible radiation^7^,^8^,^9^,^10^. And the CM enhancement can be introduced through three mechanisms: charge transfer between SERS substrate and probe molecule, molecule resonance and interfacial nonresonant interactions^11^. Many studies have demonstrated that, no matter which one dominates the enhancement, the SERS enhancement effect is sensitive to the properties of the noble metal such as the type of metal, the geometry and size of the particles, the inter space of the particles^12^,^13^. Up to now, noble metal SERS substrates with various structures such as single metal with the morphologies of nanospheres^14^, nanopyramids^15^, nanowires^16^, bimetal nano-mushrooms^17^, bimetal non-alloy nanoparticles^18^ and so on have been proposed and researched attempting to achieve high enhanced effect based on the EM. It has been demonstrated that Ag nanoparticles are superior to Au nanoparticles in terms of the SERS enhancement performance by the virtue of the strong interparticle near field coupling effect and the sharp plasmonic peak^19^,^20^. Nevertheless, the chemical stability of Ag nanoparticles is not as excellent as that of Au nanoparticles. Recently, many works have been reported to design Ag/Au hybrid (such as Au@Ag nanorod dimmers^19^, Au-Ag/Au core/shell NPs^20^ or Ag-Au-PVA thinfilm^21^) aimed at obtaining SERS substrate with ultra sensitivity and good stability. Although it is a challenge to obtain accurately uniform nanoparticles, the Ag/Au hybrids still show great promise for the SERS application.

Besides, it has also been demonstrated that by optimizing the geometry of the SERS substrate, one can achieve a further SERS enhancement using heterogeneous SERS approach^22^. One 3D SERS substrate possessing high curvature, triangularly shaped or cone-like structure serving as active sites is favorable for the heterogeneous detection due to the large field enhancement. 3D SERS substrates have great advantages in the SERS application, as they can offer large surface area, which can create more hotspots and capture more probe molecule. Therefore, multifarious 3D SERS substrates (such as multilayered colloidal crystals^23^, nanoparticle-decorated nanocanals^24^, Si modified with metal particles^25^,^26^,^27^) have been presented in recent years. Among these various 3D SERS substrates, with the attractive virtue of the high reflectivity, superior bio-compatibility, facile surface tailorability as well as the perfect multifunctionality, Si-based 3D SERS substrates are most chosen and widely employed for the SERS substrates. There are several methods to fabricate the Si-based 3D SERS substrates with large area, such as chemical method^28^, metal-assisted chemical etching^29^, laser interference lithography^27^, reactive ion etching^30^. Well-defined Si nanopattern can be produced with the method of laser interference lithography or reactive ion etching, however, these methods are expensive and time-consuming. The chemical method and metal-assisted chemical etching are relatively lost-cost and can produce a relatively uniform 3D Si with small structure, but these methods require multiple steps and multifarious strong acids are indispensable in the preparation process. Hence, a relatively simple and lost-cost technique is essential to obtain the Si-based 3D SERS substrates. What’s more, to our best known, so far, only pure metal particles associated with 3D Si substrates are studied. The Ag/Au bimetal SERS substrate, besides ultra sensitivity and good stability, can also present bi-SERS sensing properties, which can possibly be used to achieve the simultaneous detection of two different probe molecules on the single SERS substrate.

Here, in this paper, we develop a novel 3D SERS substrate based on pyramidal Si (3D-Si) decorated with bimetal Au@Ag using a simple and lost-cost method. The Au@Ag/3D-Si substrate provides uniform and high-density hot spots, which possesses the substrate ultra sensitivity. The core-shell Au@Ag enables the SERS analysis with good stability and thus improves the reproducibility of the SERS signals. The perfect SERS behaviors of the proposed Au@Ag/3D-Si substrate are also demonstrated theoretically using commercial COMSOL software.

## Experimental

**Figure 1:**
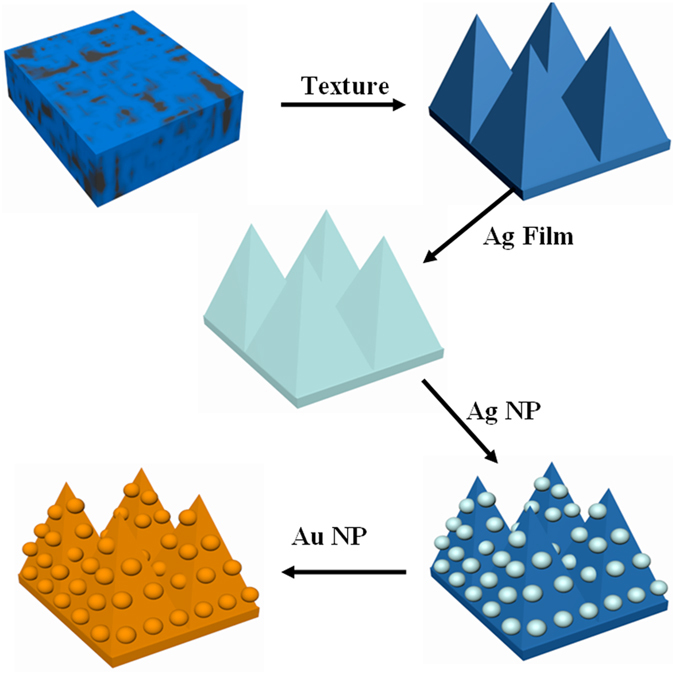
Preparation procedure of the Au@Ag/3D-Si substrate.

The preparation procedure of the Au@Ag/3D-Si substrate is elucidated in Fig. 1. 3D-Si was manufactured via a wet texturing process in NaOH solution with the aid of the anisotropic etching property of single crystal silicon. Via a thermal-evaporation method, uniform and continuous Ag film of 30 nm in thickness was deposited on the 3D-Si substrate. Then, the obtained Ag film/3D-Si substrate was annealed at 500 °C for 30 min. Next, the AgNP/3D-Si substrate was deposited by the Au film 10 nm in thickness with the ion sputtering equipment and formed Au@Ag/3D-Si substrate. As a contrast, we also prepared 3D-Si, AgNP/3D-Si (Ag/3D-Si) and Au film/3D-Si (Au/3D-Si). The 3D-Si, Ag/3D-Si and Au/3D-Si substrates were fabricated with the same method as preparing the Au@Ag/3D-Si substrate.

Scanning electron microscope (SEM, Zeiss Gemini Ultra-55) and atomic force microscopy (AFM, Bruker Multimode 8) were chosen to characterize the surface morphology of the Au@Ag/3D-Si substrate. SERS behaviors of the Au@Ag/3D-Si substrate were evaluated with a Raman microscope system (Horiba HR Evolution 800) at laser wavelength of 532 nm. The TEM image of Au@Ag nanoparticles was obtained with a transmission electron microscopy system (JEOL, JEM-2100).

## Results and Discussion

**Figure 2:**
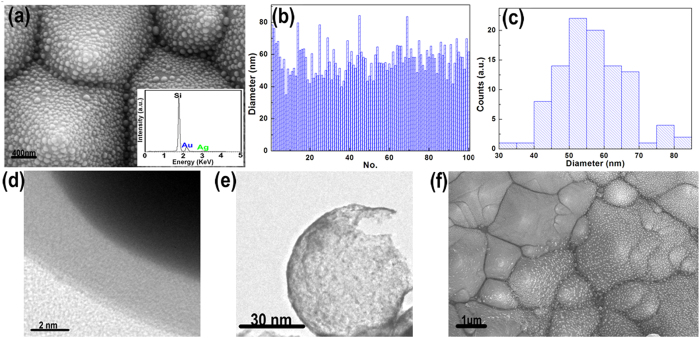
(**a**) SEM image of the Au@Ag/3D-Si sample (insert: EDX data of the Au@Ag/3D-Si). (**b**,**c**) are respectively the size and size distribution of the Au@Ag. (**d**,**e**) are TEM of the obtained Au@Ag. (**f**) SEM image of the Au/3D-Si (the left region) and Au@Ag/3D-Si (the right region).

Figure 2a presents the SEM image of the Au@Ag/3D-Si substrate. Well-ordered and uniform Au@AgNPs are observed on the 3D-Si substrate, which enables the Au@Ag/3D-Si substrate to have uniform and high-density hotspots for SERS. The size and size distribution of the Au@Ag shown in Fig. 2a are summarized in Fig. 2b,c with the assist of the image processing software Nano Measurer. Figure 2b shows the diameter of the Au@AgNPs, where we can see clearly the sizes of Au@AgNPs have a weak fluctuation. Just as the histograms shown in Fig. 2c, the size distribution of the Au@AgNPs complies with a Gaussian profile with the peak position centers at around 55 nm, which indicates that the average particle size is about 55 nm with a gap of ~10 nm. In order to give a visualized exhibition of the Au@AgNPs, we carried out TEM measurement on the Au@AgNPs which have been completely liberated from the 3D-Si substrate. Typical core-shell Au@Ag structures are present in Fig. 2d, which is much beneficial for the stability of the SERS signal. The AgNPs are fully wrapped by the Au coating layer and the thickness of the Au coating layers is ~2.5 nm. The TEM image of the core-shell Au in Fig. 2e also shows that the Au@AgNPs are spherical and the average size of the particles is ~55 nm, well consistent with the result of the SEM image. The insert in Fig. 2a shows EDX spectrum from the Au@AgNPs, where characteristic peaks associated to Au and Ag elements are detected, combined with EDX map shown in Supplementary Fig. S3, demonstrating the Au@AgNPs structure and in good agreement with the TEM results. The SEM image (Fig. 2f) exhibits us convincible evidence that AgNPs play a crucial role in the formation of the Au@AgNPs structure. The Au@AgNPs are not detected on the left region in Fig. 2f without the AgNPs acting as a medium layer, on the contrary, the Au film is obtained. This phenomenon is comprehensible, as is known, the AgNPs can act as nucleation points for the Au@AgNPs. In the region with AgNPs, the Au atoms deposit on these AgNPs and form the core-shell Au@AgNPs. While in the region without AgNPs, the atoms deposit and from the Au film due to the lack of nucleation points. This micro process of the formation of Au@AgNPs can be interpreted as the macroscopic process of snow, in the region with spherical or other shaped supports, the snowflake will fall on these supports and shape as the supports. While in the region without supports, the snowflake will fall on the ground and form a flat.

**Figure 3:**
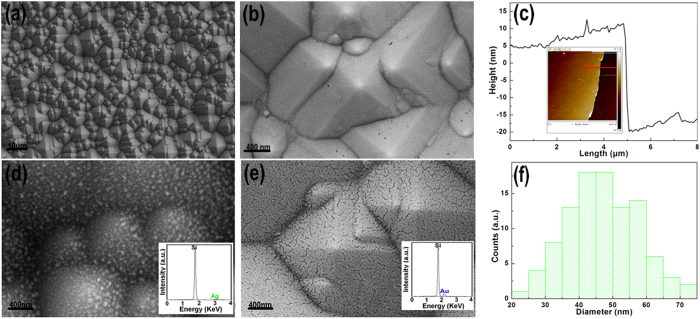
(**a**,**b**) are respectively the SEM image of the 3D-Si, Ag film/3D-Si. (**c**) AFM height image of the Ag film on 3D-Si (**d**,**e**) are respectively the SEM image of the Ag/3D-Si and Au/3D-Si sample. (**f**) Size distribution of the AgNPs on the Ag/3D-Si substrate.

To better understand the grow mechanism for the Au@Ag/3D-Si, we also prepared the 3D-Si, Ag/3D-Si and Au/3D-Si substrate. The SEM image of the 3D-Si substrate exhibited in Fig. 3a shows us clearly a typical 3D surface morphology consist of vast pyramidal silicon, where we can also observe that silicon pyramids of varying sizes coexist and distribute evenly. These varying sizes are more effectively to enhance the incident laser than that of uniform sizes and are much beneficial for the enrichment of organic molecules. The excellent behaviors of these varying sizes on the SERS will be further discussed in the section of theoretical modeling. Figure 3b shows the SEM image of the uniform Ag film/3D-Si obtained via a thermal-evaporation method. The AFM height image taken along the red line from the insert in Fig. 3c shows a step height of ~30 nm, which demonstrates that the thickness of the prepared Ag film is ~30 nm. Figure 3d exhibts the SEM image of the Ag/3D-Si, where densely aggregated AgNPs on the 3D-Si substrate after the annealing process can be seen clearly. Supplementary Fig. S2 shows the SEM image of the Ag/3D-Si under a large magnification, which give a further illustration for the Ag/3D-Si. The EDX spectrum in the insert (Fig. 3d) demonstrates the obtained AgNPs with a high purity. The average size of the AgNPs is ~50 nm with a gap of 15 nm, which can be indicated by the histogram shown in Fig. 3f,e shows the SEM image of the Au/3D-Si substrate. Just as we discussed above, only Au film is detected if AgNPs is absent. The insert in Fig. 3e shows the EDX spectrum of Au/3D-Si substrate, which demonstrates the fact that Au film is actually obtained on the 3D-Si substrate. To better illustrate the topology of 3D-Si, Au/3D-Si, Au/3D-Si and Au@Ag/3D-Si sample, AFM was carried out over 10 × 10 μm area (see Supplementary Fig. S3).

**Figure 4:**
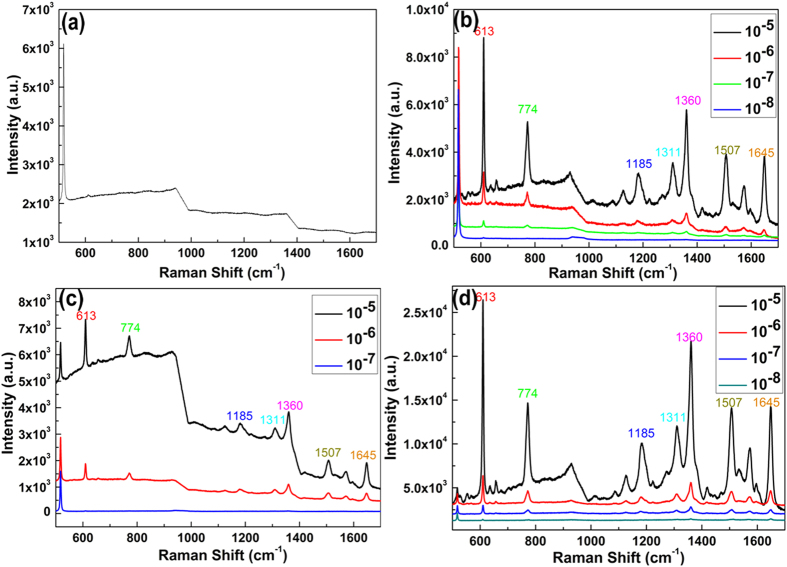
(**a**) The Raman spectra of R6G on the 3D-Si substrate with concentration of 10^−5^. (**b**) The Raman spectra of R6G on the Ag/3D-Si substrate with concentration from 10^−5^–10^−8^. (**c**) The Raman spectra of R6G on the Au/3D-Si substrate with concentration from 10^−5^–10^−7^. (**d**) The Raman spectra of R6G on the Au@Ag/3D-Si substrate with concentration from 10^−5^–10^−8^.

To estimate the SERS activity of Au@Ag/3D-Si substrate, we compared the behaviors of Au@Ag/3D-Si substrate with that of 3D-Si, Ag/3D-Si and Au/3D-Si substrate. The R6G molecules with varied concentrations were chosen as the probe molecule and all the SERS spectra were implemented on the same conditions. For the case of 3D-Si substrate, the SERS activity is poor where only weaker peaks of R6G with concentration of 10^−5^ are detected and with the decrease of the concentration, the SERS spectra can be hardly detected (Fig. 4a). The poor SERS activity is introduced by the lack of surface plasmons excited between the metal. In the cases of Ag/3D-Si, Au/3D-Si and Au@Ag/3D-Si substrates, the enhancement of the Raman signal is immensely improved and the peaks located at 613, 774, 1185, 1311, 1360, 1507 and 1645 cm^−1^ are well consist with the previous reports for R6G^7^. As shown in Fig. 4b–d, the SERS spectra for R6G with concentration from 10^−5^–10^−7^ can be easily observed on all the Ag/3D-Si, Au/3D-Si and Au@Ag/3D-Si substrates, while the intensities of the characteristic peaks is varied on different substrates. The enhancement factor (EF) for Au@Ag/3D-Si substrates was calculated according to the following equation


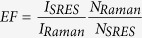


where *I*_SERS_ and *I*_Raman_ are respectively the intensity of the SERS band and the normal Raman spectra, and *N*_Raman_ and *N*_SERS_ are respectively the numbers of R6G molecules illuminated by the laser spot. Therefore, we can calculate that the EF for the Au@Ag/3D-Si substrate is 1.2 × 10^9^, which is 6.3 × 10^2^ times larger than that on pure AgNP array^31^, 4.22 times larger than that with Ag-Au-PVA thin film^32^ and is comparable with that on Au/Graphene/Ag^33^.

Supplementary Table S1 shows the enhancement on 613, 774, 1360 cm^−1^ versus substrates. The intensities of SERS spectra of R6G with concentration of 10^−5^ on Au@Ag/3D-Si are 4.7–6 times stronger than that of Ag/3D-Si, 12–16 times stronger than that of Au/3D-Si. Similar results are also obtained with different concentrations. These enhancement differences can be ascribed to the diverse surface morphology and dominated enhanced mechanism for different substrates. Using 3D-Si as SERS substrate, as the absence of metal nanaparticles, the EM can not work and only enhancement for the incident laser is achieved. Therefore, in this case, the obtained Raman signal is relatively weaker and is considered as the conventional Raman signal. Compared with the 3D-Si substrate, the SERS signals of Ag/3D-Si are ~80 times larger than that of 3D-Si substrate and the SERS signals of Au/3D-Si are ~25 times larger than that of 3D-Si substrate. These huge improvements for the enhancement are introduced by the existence of the metal nanoparticles or film. What should be noted here is that the enhancement of Ag/3D-Si and Au/3D-Si is different, a larger one is acquire for the former. There are two main reasons for this different: (1) compared with metal film, metal nanoparticles are more suitable for the EM as surface plasmons can be excited between metal particles. (2) Ag is superior to Au in terms of the SERS enhancement performance by the virtue of the strong interparticle near field coupling effect and the sharp plasmonic peak. The additional improvement for the SERS signal on Au@Ag/3D-Si can be assigned to the core-shell bimetal strcuture, where the electronic field can be significantly enhanced via introducing the Au shell coating on the AgNPs. The more detailed discussion for these varied enhancements will be further provided in the section of theoretical modeling.

**Figure 5:**
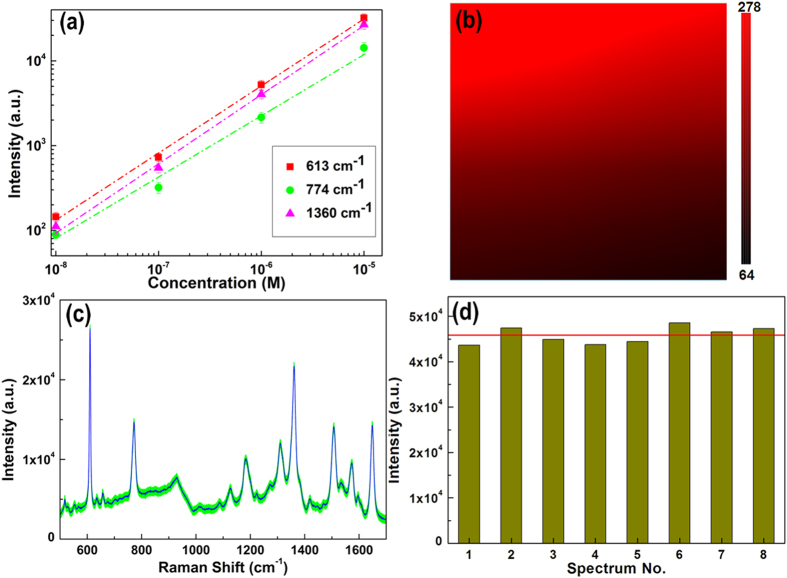
(**a**) Raman intensity of R6G peaks at 613, 774 and 1360 cm^−1^ as a function of the molecular concentration on the Au@Ag/3D-Si substrate, in log scale. (**b**) SERS map of the R6G peaks at 613 cm^−1^ with concentration of 10^−8 ^M on the Au@Ag/3D-Si substrate over 20 × 20 μm area. (**c**) Average SERS spectrum of the R6G from 8 positions on a same Au@Ag/3D-Si substrate (blue line). (**d**) Intensity distribution of the peak at 613 cm^−1^ from 8 different batches of Au@Ag/3D-Si substrates.

To investigate the ability for the quantitative detection of R6G, the linear fit curves are illustrated in Fig. 5a. It is thoroughly distinct that reasonable linear response in log scale is achieved between the intensity of SERS signal and the R6G concentration with concentration from 10^−5^ to 10^−8 ^M. In addition to the SERS activity and the ability for the quantitative detection, the homogeneity and the reproducibility for the SERS signals are also crucial parameters for the SERS application as a routine and sensitive analytical tool. We measured the SERS map of the R6G peaks at 613 cm^−1^ with concentration of 10^−8^ M over 20 × 20 μm area with a step of 1 μm to estimate the homogeneity of SERS signals on the Au@Ag/3D-Si substrate. The relatively smooth and uniform color distribution in the SERS map (Fig. 5b) with only a little dark region indicates that the prepared Au@Ag/3D-Si substrate possesses well homogeneity for SERS signal. To further demonstrate the perfect homogeneity of the Au@Ag/3D-Si, we collected the SERS signals randomly from locations with the interval of above 2 mm. The SERS spectra of the R6G from 8 positions on a same Au@Ag/3D-Si substrate are shown in Fig. 5c. The SERS signals from these 8 positions overlap and form a narrow shadow region marked with green, which indicates the fact that the SERS signals fluctuate mildly for various peaks. The average spectrum of the R6G from 8 positions is marked by the blue line. Based on these results, we can draw a conclusion that, even with a millimeter-scale span, the homogeneity of the Au@Ag/3D-Si is still excellent, presenting broad prospects for practical SERS application. To evaluate the reproducibility for the SERS signals, SERS spectra of the R6G from 8 different batches of Au@Ag/3D-Si substrates were collected. Figure 5d shows the intensity distribution of the peak at 613 cm^−1^ of R6G with concentration of 10^−5^ M from 8 different batches of Au@Ag/3D-Si substrates. The red line in Fig. 5d is the average value of the intensities for the peak at 613 cm^−1^ from these 8 different batches of Au@Ag/3D-Si substrates. It is obvious that all the data fluctuates mildly around the average value, lying within ±6% variation range. The teeny deviation from substrate to substrate indicates the reproducibility of our proposed Au@Ag/3D-Si SERS substrates is remarkable and can meet the requirements for practical SERS application. The remarkable reproducibility of our proposed Au@Ag/3D-Si SERS substrates can be assigned to two aspects: first, the facile reproduction of the Au@Ag/3D-Si SERS substrates can effectively reduce the influence from the substrate; second, the uniform and porous structure of 3D-Si substrate can homogenously and effectively capture the probe molecules.

**Figure 6:**
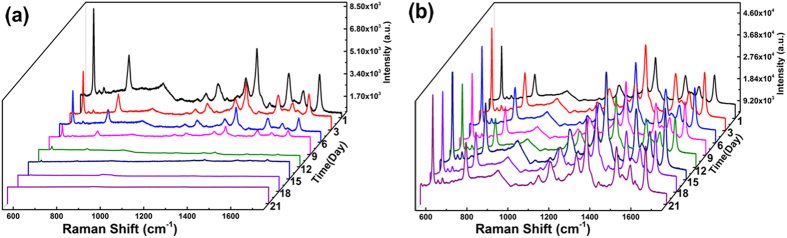
Stability of the SERS signal of R6G with concentration of 10^−5 ^M on (**a**) Ag/3D-Si substrate, and on (**b**) Au@Ag/3D-Si SERS substrate.

It is noticed that the stability of the SERS signal is also crucial for the practical application. Therefore, we further measured the stability of the Au@Ag/3D-Si substrate every three days in the humid environment. As a comparison, the stability of the Ag/3D-Si substrate was also detected. Just as expected, compared with that of the Ag/3D-Si substrate, the stability of the Au@Ag/3D-Si substrate is improved. As exhibited in Fig. 6, the SERS signals of R6G on the Au@Ag/3D-Si substrate maintain considerably stable, while that on the Ag/3D-Si substrate decreases quickly and obviously. Because of the chemical stability of the Au, the core-shell Au structure can isolate AgNPs from surrounding humid environment and act as passivation layer to suppress the surface oxidation of the AgNPs. Consequently, our proposed Au@Ag/3D-Si substrate is advantageous in terms of the long-time stability.

**Figure 7:**
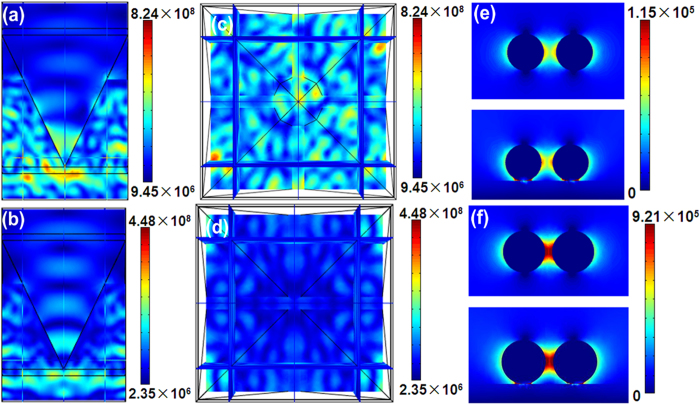
(**a**,**c**) are respectively the y-z and x-y views of the electric field distribution on the 3D-Si sample inserted with a small pyramid. (**b,d**) are respectively the y-z and x-y views of the electric field distribution on the 3D-Si sample. (**e**) the x-y view (top) and the y-z view (bottom) of the electric field distribution on the 50nm Ag/Si with the gap of 15 nm. (**f**) The x-y view (top) and the y-z view (bottom) of the electric field distribution on the 55 nm Au@Ag/Si with the gap of 10 nm.

In order to further indentify and better understand the perfect SERS behaviors of the Au@Ag/3D-Si substrate, we calculated and analyzed the local electric field properties using commercial COMSOL software. As the large size difference of the 3D-Si and Au@Ag, we modeled the electric field properties of 3D-Si and Au@Ag structure respectively to investigate the SERS behavior of the Au@Ag/3D-Si substrate. We built the theoretical model as the structure shown in Fig. 1, where a small pyramid was insert in the middle region and set the size of the pyramid according to the actual shape as shown in Fig. 2. Figure 7a,c respectively show the y-z and x-y views of the electric field distribution on the 3D-Si inserted with a small pyramid irradiated by a plane wave of 532 nm (lying in the absorption band of the Au@Ag/3D-Si substrate as shown in Supplementary Fig. S4). It can be seen clearly that, compared with that of 3D-Si without the inserted small pyramid (Fig. 7b,d), the local electric field of the incident light is magnified strongly on the 3D-Si with the inserted small pyramid. The intensity for the local electric field on the 3D-Si with the inserted small pyramid is ~2 times stronger than that of 3D-Si without the inserted small pyramid. Although just the incident laser amplification is next-to-no benefit for the SERS, it will be of tremendous assistance to the SERS behaviors of the Au@Ag/3D-Si substrate. Combined with the 3D-Si substrate, the magnitude of electrical field for the plasmonic resonance between Au@AgNPs will be obviously increased with the assist of the incident laser amplification. It is noted that the electric field distribution on the 3D-Si sample with an inserted small pyramid is relatively uniform, which makes the Au@AgNPs/3D-Si possess perfect homogeneous SERS signal. To investigate the superiority of the Au@AgNPs to AgNPs in the aspect of SERS, we respectively calculated the electric field distributions of the Au@AgNPs and AgNPs. The radius of Au@AgNPs was set as 27.5 nm including a 2.5 nm Au core-shell coating with a gap of 10 nm and the radius of AgNPs was set as 25 nm with a gap of 15 nm, in accordance with the experimental results. Just as our expects, the intensity for the local electric field on the Au@AgNPs is ~8 times stronger than that of AgNPs, which is well consistent with the experimental results (4.7–6 times stronger). Based on these theoretical results, the Au@AgNPs/3D-Si SERS substrate with higher sensitivity will be realized by further optimizing its structure.

In conclusion, we have offered a novel and efficient SERS based on Au@Ag/3D-Si. By combining the merits of SERS activity of Ag, the chemical stability of Au and the large field enhancement of 3D-Si, the Au@Ag/3D-Si substrate exhibits sensitivity, homogeneity, reproducibility and chemical stability. Using R6G as probe molecule, the SERS results imply that the Au@Ag/3D-Si substrate is superior to the 3D-Si, Ag/3D-Si and Au/3D-Si substrate. These SERS behaviors are also confirmed in theory via a commercial COMSOL software. The corresponding experimental and theoretical results suggest that our proposed Au@Ag/3D-Si substrate is expected to offer a new and practical way to accelerate the development of label-free SERS detection.

## Additional Information

**How to cite this article**: Zhang, C. *et al.* Gold@silver bimetal nanoparticles/pyramidal silicon 3D substrate with high reproducibility for high-performance SERS. *Sci. Rep.* **6**, 25243; doi: 10.1038/srep25243 (2016).

## Supplementary Material

Supplementary Information
