# Supplementary material for: Gold@silver bimetal nanoparticles/pyramidal silicon 3D substrate with high reproducibility for high-performance SERS
Source: Sci Rep. 2016 May 4;6:25243. doi: 10.1038/srep25243 (PMC4855179; doi:10.1038/srep25243)
Supplement: Supplementary Information [file srep25243-s1.doc]

Gold@silver bimetal nanoparticles/pyramidal silicon 3D substrate with high reproducibility for high-performance SERS

**Chao Zhang,^1^ Shou Zhen Jiang,^1,2^ Cheng Yang, ^1^ Chong Hui Li,^1^ Yan Yan Huo,^1^ Xiao Yun Liu,^1^ Ai Hua Liu,^1^ Qin Wei,^1^ Sai Sai Gao,^1^ Xing Guo Gao,^3^ and Bao Yuan Man^1*^**

*^1^**School of Physics and Electronics, Shandong Normal University, Jinan 250014,* *China*

*^2^State Key Lab of Crystal Materials Shandong University, Jinan 250100, China*

*^3^School of Science, Qilu University of Technology, Jinan 250353, China*

*^*^ Corresponding author*: *byman@sdnu.edu.cn*


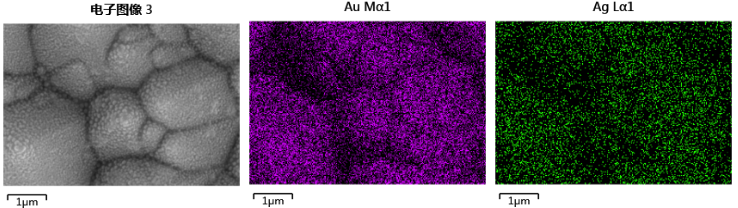


**Supplementary Figure S1. SEM** and EDX map of the Au@Ag/3D-Si sample


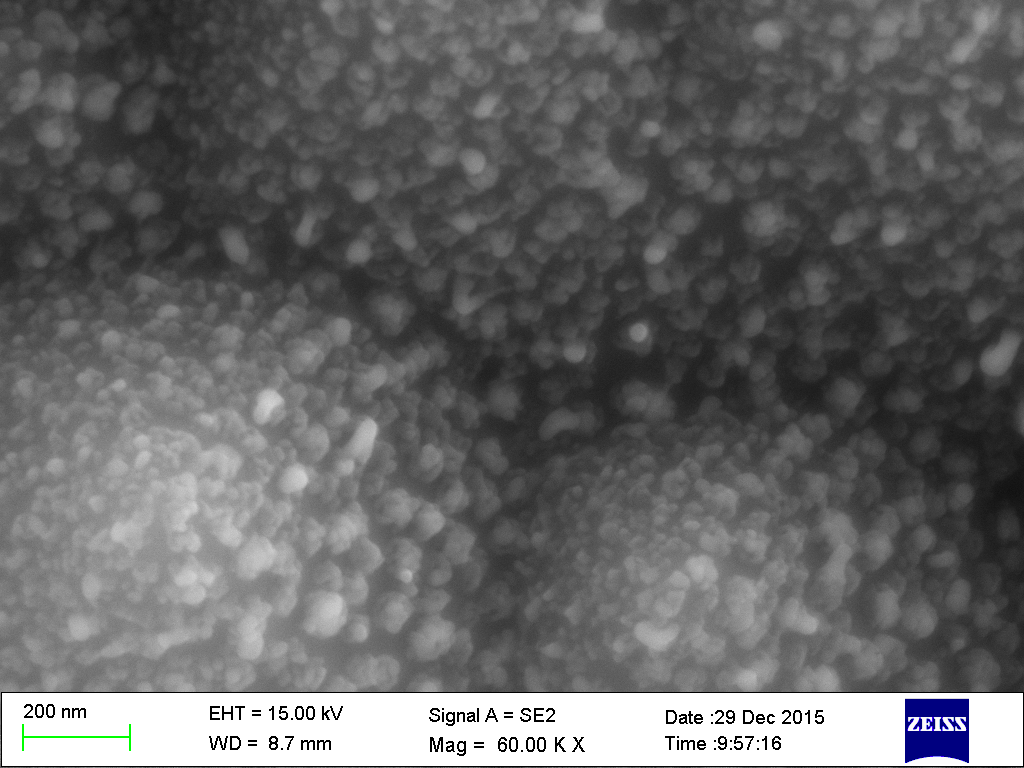


**Supplementary Figure S2. SEM** image of the Ag/3D-Si sample under a large magnification.


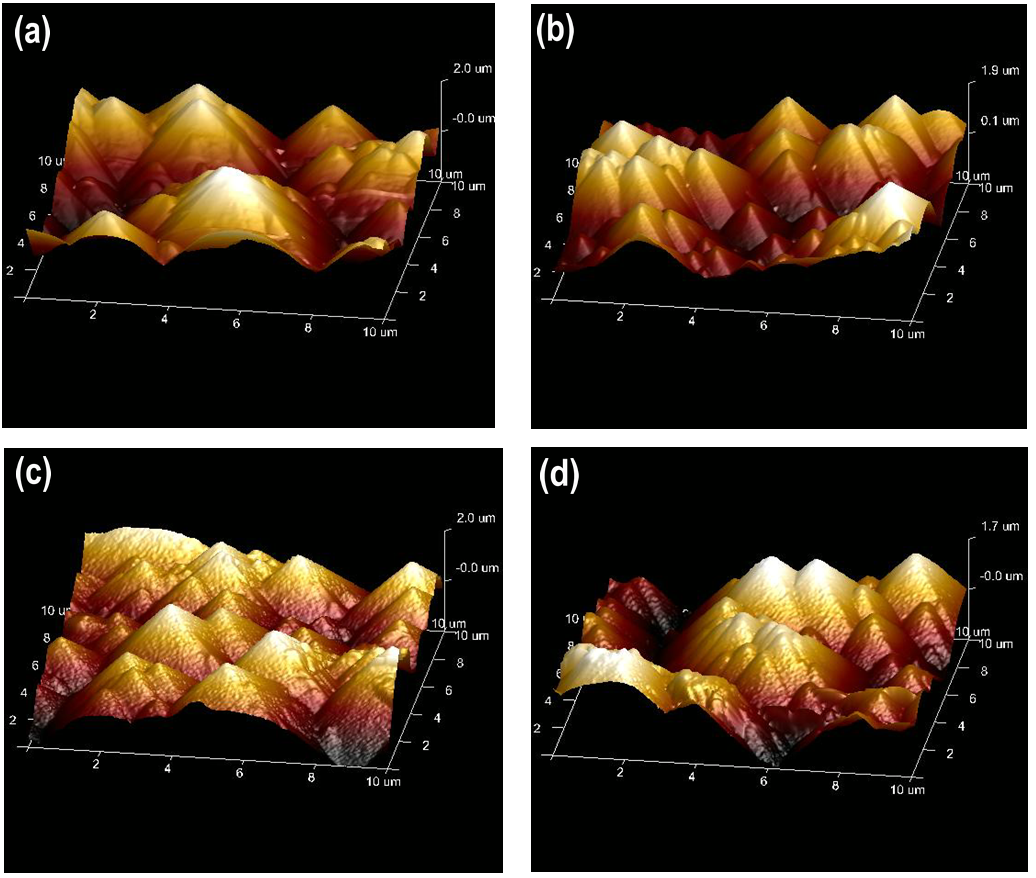


**Supplementary Figure S3. (a)-(d)** are respectively the AFM image of the 3D-Si, Au/3D-Si, Au/3D-Si and Au@Ag/3D-Si sample over 10×10μm area.


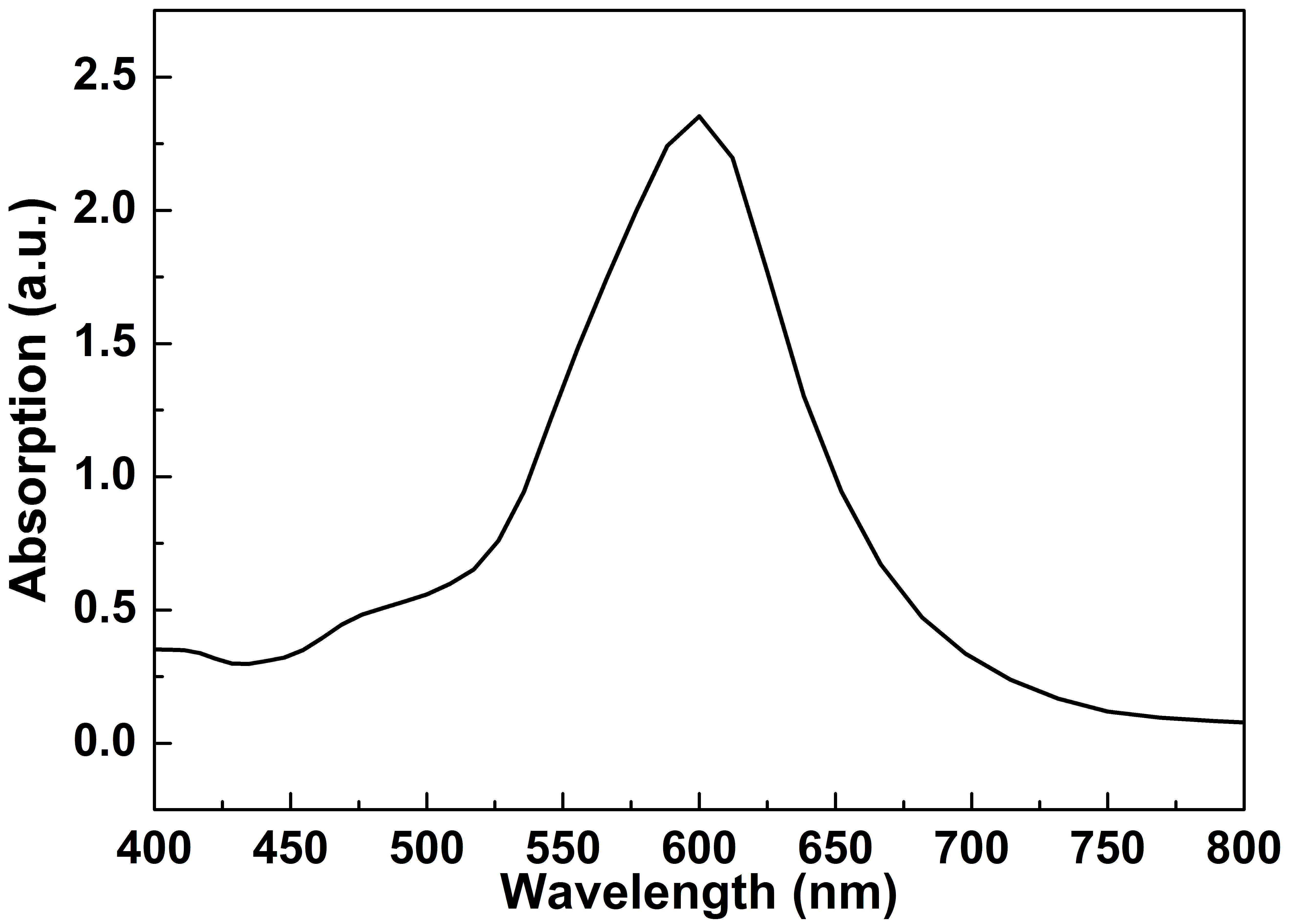


**Supplementary Figure S4. The** optical absorption spectrum of the Au@Ag/3D-Si substrate.

| Peak (cm^-1^) | Substrate | Concentration (10^-5^M) | Concentration (10^-6^M) | Concentration (10^-7^M) | Concentration (10^-8^M) |
| --- | --- | --- | --- | --- | --- |
| 613 | 3D-Si | 80 |  |  |  |
|  | Au/3D-Si | 1978 | 724 | 37 |  |
|  | Ag/ 3D-Si | 6718 | 1466 | 318 | 60 |
|  | Au@Ag/ 3D-Si | 32014 | 5215 | 726 | 144 |
| 774 | 3D-Si | negligible |  |  |  |
|  | Au/3D-Si | 857 | 350 | 18 |  |
|  | Ag/ 3D-Si | 2711 | 634 | 221 | 37 |
|  | Au@Ag/ 3D-Si | 14200 | 2150 | 320 | 90 |
| 1360 | 3D-Si | negligible |  |  |  |
|  | Au/3D-Si | 2464 | 637 | 39 |  |
|  | Ag/ 3D-Si | 4544 | 934 | 244 | 47 |
|  | Au@Ag/ 3D-Si | 26812 | 4025 | 546 | 111 |

**Supplementary Table S1. The enhancement on 613, 774, 1360cm^−1^ versus substrates**
